# Effect Modification by Baseline Mortality in the MORDOR Azithromycin Trial

**DOI:** 10.4269/ajtmh.18-1004

**Published:** 2019-02-07

**Authors:** Assaf P. Oron, Roy Burstein, Laina D. Mercer, Ahmed M. Arzika, Khumbo Kalua, Zakayo Mrango, Sheila K. West, Robin L. Bailey, Travis C. Porco, Thomas M. Lietman

**Affiliations:** 1 Institute for Disease Modeling, Bellevue, Washington;; 2 Institute for Health Metrics and Evaluation, University of Washington, Seattle, Washington;; 3 The Carter Center, Niamey, Niger;; 4 Blantyre Institute for Community Outreach and the College of Medicine, University of Malawi, Blantyre;; 5 National Institute for Medical Research, Dar es Salaam, Tanzania;; 6 The Dana Center, Johns Hopkins University School of Medicine, Baltimore, Maryland;; 7 The London School of Tropical Hygiene and Medicine, London, United Kingdom;; 8 Francis I Proctor Foundation, University of California, San Francisco, San Francisco, California;; 9 Department of Ophthalmology, University of California, San Francisco, San Francisco, California

## Abstract

We examined whether baseline mortality risk, as a function of child age and site, modified the azithromycin mortality-reduction effect in the *Macrolide Oraux pour Réduire les Décès avec un Oeil sur la Résistance* (MORDOR) clinical trial. We used the Cox proportional hazards model with an interaction term. Three models were examined representing three sources for the baseline-risk covariate: two using sources external to MORDOR and the third leveraging data within MORDOR. All three models provided moderate evidence for the effect becoming stronger with increasing baseline mortality (*P* = 0.02, 0.02, and 0.07, respectively) at the rate of approximately 6–12% additional mortality reduction per doubling of baseline mortality. Etiological and programmatic implications of these findings are discussed.

## INTRODUCTION

The Macrolide Oraux pour Réduire les Décès avec un Oeil sur la Résistance (MORDOR)† clinical trial conducted in Malawi, Niger, and Tanzania provided significant evidence for reduction in child mortality in sub-Saharan Africa, following biannual mass administration of azithromycin.^1^ This evidence has been further corroborated by a recent meta-analysis that included data from two additional clinical trials, although MORDOR accounted for the vast majority of person-years (py) and observed death events.^2^

Across these trials and in MORDOR in particular, there were intriguing patterns that suggested mortality reduction might be disproportionately concentrated in populations with higher baseline mortality. MORDOR was powered for some subgroup analyses: the Niger site had by far the highest mortality rate, the strongest effect (18% mortality reduction, versus the trial’s overall average estimate of 13.5%), and the only significant single-site effect. Similarly, the youngest age group (1–5 months old at treatment) suffered the highest baseline mortality rate and also saw the strongest and most significant mortality reduction (25%, averaged across all sites), followed by the second youngest group (6–11 months at treatment, 14% reduction). However, the trial was not powered to detect effect modification, and indeed the interactions between effect strength and either site or age were not significant.

The question whether effect magnitude is related to geographic and demographic factors, or to some other baseline risk profile that incorporates such factors, has substantial implications for planning future azithromycin interventions. Therefore, we reanalyzed MORDOR data to examine whether the baseline mortality rate, rather than age or site alone, is associated with effect magnitude. Whereas the main MORDOR analysis was conducted at the randomization-cluster (“village”) level, our analysis used individual survival histories. To test sensitivity and robustness of potential effect modification, we examined three different sources of data for baseline mortality. Two of the sources were estimates external to MORDOR and one was from within the study’s data.

## METHODS

### Data source.

A redacted and de-identified version of MORDOR data was prepared by T. C. P. MORDOR study properties, and its main results were reported elsewhere.^1^ MORDOR was approved by the Committee on Human Research at the University of California, San Francisco; the Institutional Review Boards at Emory University; the College of Medicine, the University of Malawi, Blantyre; the Niger Ministry of Health; the Tanzanian National Institute for Medical Research; the London School of Hygiene and Tropical Medicine; and the Johns Hopkins University School of Medicine. Secondary analyses such as the present one were covered by these approvals. Jittered coordinates were provided for each individual entry, but we used them only to calculate median cluster coordinates, retaining individual de-identifiability throughout the rest of the analysis.

### Dataset preparation.

The study consisted of four consecutive follow-up phases, each lasting approximately 6 months on average. Individual child key, age at phase start, visit dates (at which treatment or placebo was administered), and days in follow-up for each phase were provided. Constructing self-consistent individual histories was thus possible, but required some straightforward data reconciliation, which affected ∼6% of children. We provide further details in the Supplement.

### Baseline mortality covariates.

The first source for baseline-mortality estimates was standard national cluster-sample surveys: the Demographic and Health Survey (DHS) for Malawi 2015–2016 and Tanzania 2015–2016, and the Multiple Indicator Cluster Survey (MICS) for Niger, 2012.^3–5^ We used survey estimates specific to the subnational regions where MORDOR took place (Mangochi in Malawi, Dosso in Niger, and Eastern Region in Tanzania). Because the Niger MICS was 3 years older and that survey showed a 5–6% per year mortality reduction during the years leading up to 2012, we reduced Niger’s numbers by 15% (a sensitivity analysis with no reduction showed similar overall results). Rates and standard errors were available for the 1- to 11-month-age and 12- to 59-month-age bins.

The second source provided by R. B. uses methods published elsewhere: 1,000 posterior draws of child mortality probability estimates from the Institute of Health Metrics and Evaluation (IHME) Local Burden of Disease project on a 5 × 5-km grid for a region covering each site.^6^ Mortality estimates were available separately for 1- to 11-, 12- to 35-, and 36- to 59-month-age bins. For each study site and age bin, cluster-median coordinates were matched to the closest grid cell, and averages (logit-transformed, which was the IHME model scale) weighted by cluster population were calculated for each posterior draw.

**Figure 1.**
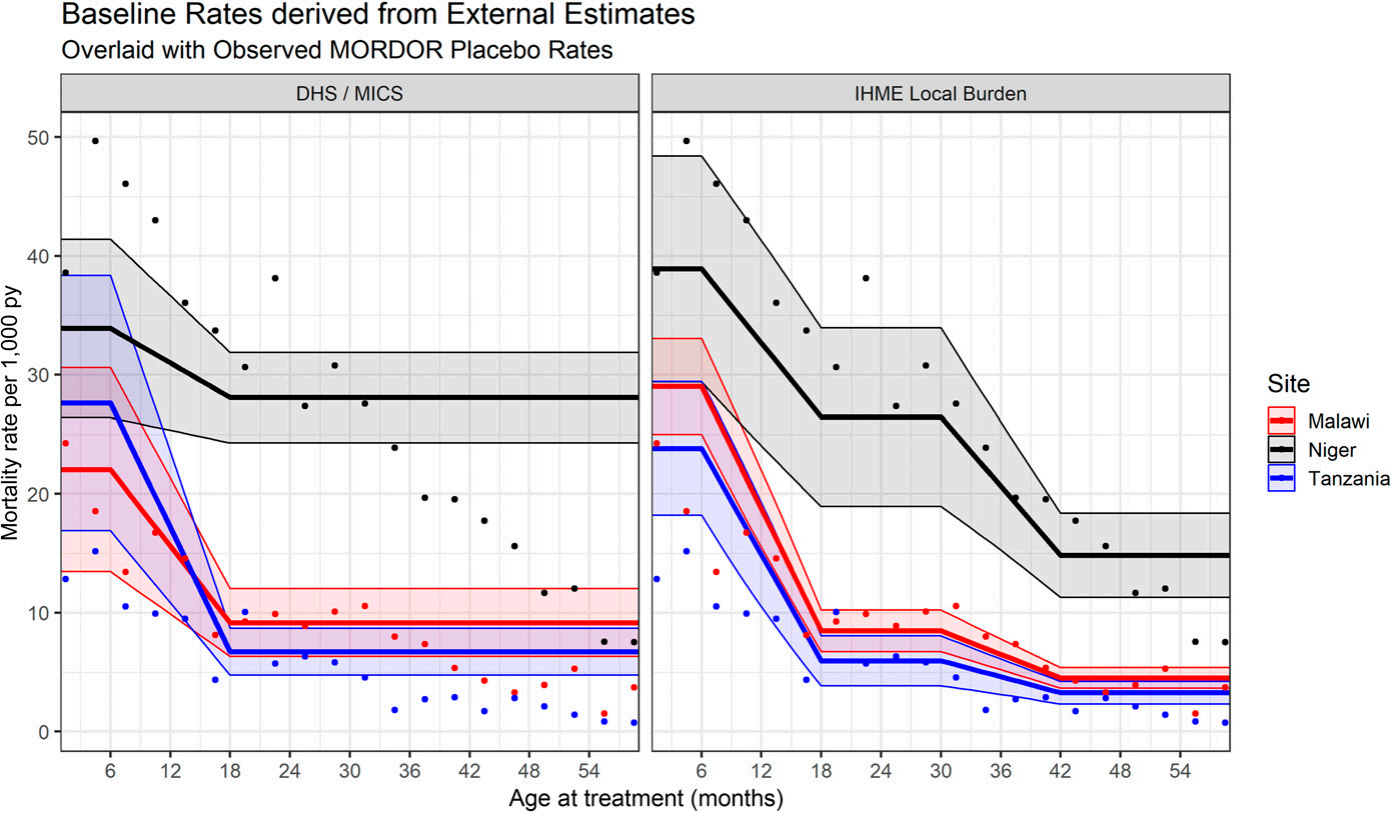
Externally derived baseline mortality curves used as risk covariates in the model. Left: Demographic and Health Survey (DHS) and Multiple Indicator Cluster Survey (MICS) estimates. Right: Institute of Health Metrics and Evaluation (IHME) local burden estimates. The bands indicate 95% CIs. The dots in both panes indicate crude observed mortality rates in Macrolide Oraux pour Réduire les Décès avec un Oeil sur la Résistance (MORDOR) placebo arm, calculated by site and 3-month-age bins. This figure appears in color at www.ajtmh.org.

With both sets of external estimates, a gradual transition between discrete age bins is warranted, for several reasons. Because the average phase duration was 6 months, most children treated at 6–11 months of age (and also, 30–35 months in the IHME case) matured into the next age bin during the phase. In addition, a dramatic, instantaneous drop in mortality risk at exactly 12 months is unrealistic. Last, there was strong “heaping” of reported ages every 6 months, in particular around 12 months of age. Therefore, rather than using two to three constant mortality rates per site, we linearly interpolated mortality rates within 6 months of age bin boundaries (between 6 and 18 months of age, and also between 30 and 42 months for the IHME estimates; see Figure 1).

The third set of baseline mortality estimates was derived from MORDOR data, by estimating survival as a smooth function of treatment age separately for each site and phase, averaged across both arms, via generalized additive models with a Cox survival link (R package mgcv).

Uncertainty of baseline-mortality estimates in the models, regardless of source, was accounted for by assuming they were missing and using standard imputation techniques guided by each source’s standard errors.^7^ We used *B* = 400 imputations for all models.

### Statistical model.

For each baseline-mortality data source, we fitted a separate Cox proportional hazards regression model with a time-varying baseline-mortality covariate (i.e., a different covariate value for each child–phase combination). The effect of interest was interaction between treatment arm and baseline mortality. Symbolically,

$$\log\left\{{\text{λ}}_{ip}\left(\text{age};s,p\right)\right\}=\log\left\{{\text{λ}}_{0}\left(\text{age};s,p\right)\right\}+{\text{β}}_{a}a_{i}+{\text{β}}_{R}\text{log}R\left(s,T{\text{age}}_{i}\right)+{\text{β}}_{\text{mod}}a_{i}\text{*log}R\left(s,T{\text{age}}_{i}\right),$$

where *i*, *s*, *a*, and *p* are the individual, site, arm, and phase indicators, respectively; λ_0_ and λ_*ip*_ are the baseline mortality hazard (stratified by site and phase) and individual *i*’s specific hazard curve during phase *p*, respectively; log*R* is the logarithm of the baseline-mortality covariate described previously (transformed to match the Cox model’s log-hazard scale); and age and *T*age are continuous age (used as the model’s universal “time counter”) and age at treatment, which remains fixed over time for each individual–phase combination. Finally, β_*a*_, β_*R*_, and β_mod_ are the effect sizes of the azithromycin (AZM) arm, baseline mortality, and their interaction, respectively. Robust standard errors were calculated, to account for observation clustering by randomization unit.

The baseline mortality predictor was centered around 15 deaths per 1,000 py, roughly equivalent to under-5 mortality probability of 100 per 1,000 live births, assuming neonatal mortality probability of 25–30 per 1,000 live births, typical to most of sub-Saharan Africa. This rate is also close to the overall mean mortality rate observed during MORDOR. For inference, we used the classical asymptotic test on the interaction term’s imputation t-statistic. Given the post hoc nature of our analysis, we considered *P* < 0.01 as significant, whereas 0.01 ≤ *P* < 0.1 was accorded the intermediate status of indicative evidence. All analyses were performed by A. P. O. using R (The R Foundation for Statistical Computing, Vienna, Austria). Additional model details appear in the Supplement.

## RESULTS

The individually reconciled dataset follows 270,229 child histories over 337,966 py, with 4,990 mortality events across the three sites: 72% of them in Niger, 21% in Malawi, and 7% in Tanzania. Figure 1 shows mortality rates observed on the placebo arm in each site separately, binned by 3-month age intervals (dots). In Niger and Tanzania, mortality rates peaked among infants treated during the second quarter of life, and in Malawi, they were worst among the youngest infants. Rates decreased dramatically after the first birthday, but less so in Niger, which had the highest mortality rates at all age bins. The IHME-derived baseline mortality estimates (right, solid lines with uncertainty bands) show markedly better agreement with observed MORDOR patterns than the DHS/MICS-derived lines (left).

**Figure 2.**
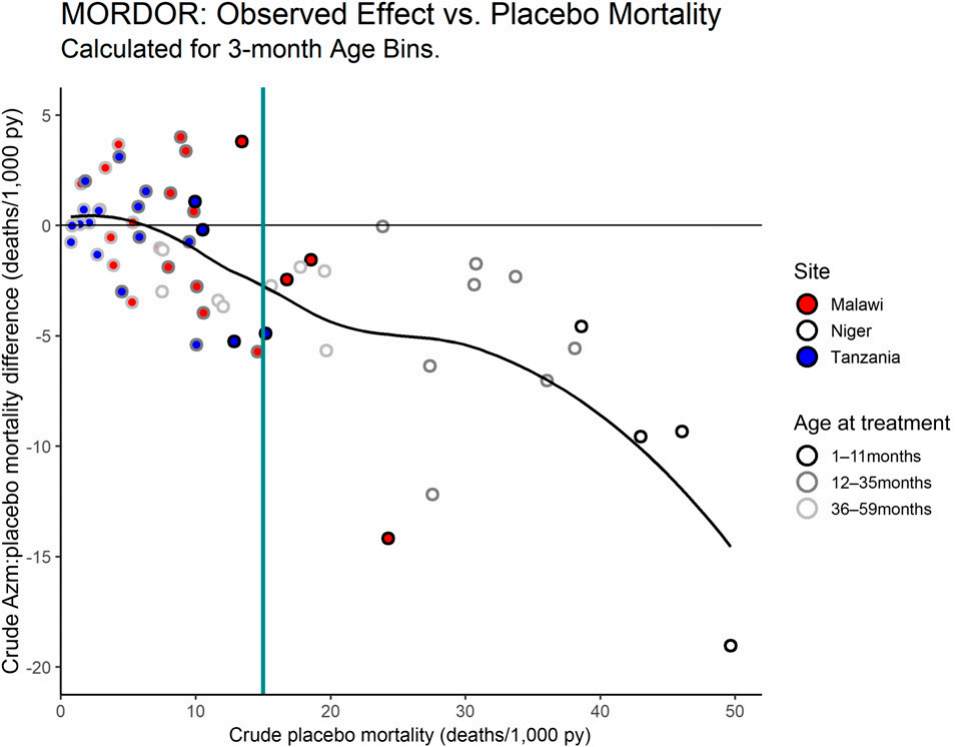
Observed mortality rates calculated by site and 3-month-age bins: placebo rates (*x* axis) vs. the difference between the arms (*y* axis). Negative difference indicates net mortality reduction on the azithromycin arm. Site is indicated by circle color and age group by the circle outlines’ shade of gray. The solid black curve is an empirical locally estimated scatterplot smoothing (LOESS) fit using span parameter 0.75. Compare with ref. 2, Fig. 2. This figure appears in color at www.ajtmh.org.

To provide a perspective on effect modification directly from the raw data, Figure 2 shows the placebo rates from Figure 1 versus the rate differences between the arms. A perfectly proportional azithromycin effect (i.e., no effect modification) would trace a diagonal linear pattern from the origin. By contrast, the observed pattern does not substantially depart from zero effect until a mortality rate of ∼10 deaths/1,000 py. At the high-mortality end, the effect increases nonlinearly. The latter pattern is driven by Niger data only, as this was the only site with observed age-bin placebo mortality rates greater than 25 deaths/1,000 py. However, observations from all three sites overlap in the range of 10–25 deaths/1,000 py, where they all seem to follow a similar trend.

**Figure 3.**
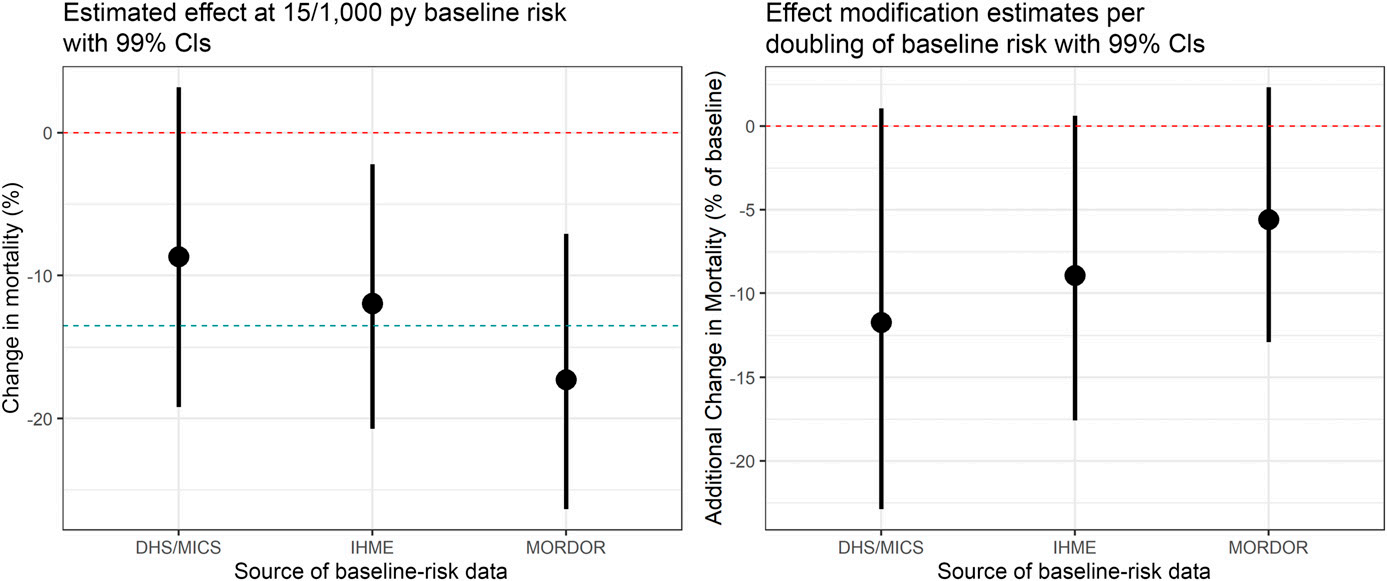
Estimates from the effect-modification models with 99% CIs. Left: the main effect at a baseline risk of 15 deaths per 1,000 py. The blue line marks the overall Macrolide Oraux pour Réduire les Décès avec un Oeil sur la Résistance (MORDOR) estimate of 13.5% mortality reduction. Right: effect modification by baseline risk, represented as additional mortality reduction per doubling of baseline risk. This figure appears in color at www.ajtmh.org.

Figure 3 shows the estimates and 99% CIs for effect modification in the full models, using the three sources of baseline-mortality rates (right), as well as the estimated effect at the reference baseline risk of 15 deaths per 1,000 py (left). All models indicate some enhancement of the azithromycin effect with increasing baseline risk; an enhancement that is indicative, yet not significant at α = 0.01. Specifically, the DHS/MICS-informed model suggests 11.7% additional mortality reduction as baseline risk is doubled (99% CI: −22.9%,+1.1%; *P* = 0.02), the IHME-informed model indicates 8.9% additional reduction (99% CI: −17.6%,+0.6%; *P* = 0.02), and the model using estimates derived from MORDOR data indicated 5.6% additional reduction per doubling (99% CI: −12.9%,+2.3%; *P* = 0.07).

Azithromycin effect estimates at the reference rate of 15/1,000 py (Figure 3, left) show the reverse order, with the DHS/MICS model indiating a relatively weak effect at this baseline rate (8.7% reduction, 99% CI: −3.2%,19.2%; *P* = 0.06), the IHME model significant and close to the mean MORDOR effect (12.0% reduction, 99% CI: 2.2%,20.7%; *P* = 0.002), and the MORDOR-derived model strongest (17.3% reduction, 99% CI: 7.1%,26.3%; *P* < 0.001).

**Figure 4.**
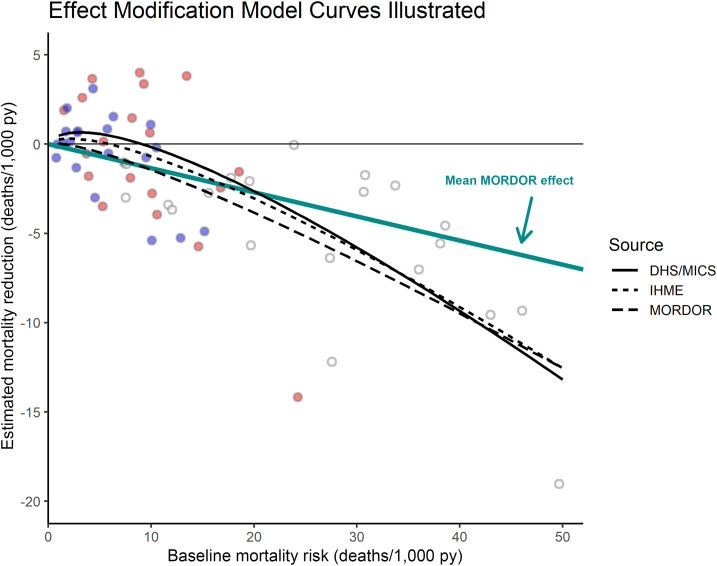
Fitted effect-size curves from the three interaction models. The diagonal blue line indicates the average Macrolide Oraux pour Réduire les Décès avec un Oeil sur la Résistance (MORDOR) effect with no interaction. The age-bin observations from Figure 2 are added in the background as visual reference. This figure appears in color at www.ajtmh.org.

Figure 4 illustrates the three estimated model curves as a function of baseline mortality. The externally informed models trace similar trajectories, slightly detrimental (but certainly not significant) at very low mortality rates, remaining weaker than the average MORDOR estimate of 13.5% mortality reduction (diagonal blue line) until ∼20 deaths/1,000 py, and becoming stronger after that. The MORDOR-derived curve is closer to linear and shows a stronger effect almost throughout.

## DISCUSSION

A concurrent article examines the baseline-mortality effect modification question using a complementary approach, namely, splitting each MORDOR study region into rectangles.^8^ That article’s regression model is fitted at the subregional-summary level rather than using individual histories, and also incorporates all studies used in Oldenburg et al.’s^2^ meta-analysis. Its baseline-mortality variable is most closely related to our model that uses within-MORDOR mortality estimates. It implicitly assumes that the effect is age-agnostic (except, to a limited extent, in their sensitivity analysis adjusting for median age), whereas our article implicitly assumes that baseline mortality is identical for same-age children within each site. Despite these differences, when restricted to MORDOR data only, the other article finds evidence at a significance level similar to ours (*P* = 0.04), but on addition of data from two much smaller studies, the evidence becomes substantially weaker (*P* = 0.12), mostly because of one of those studies^9^ showing a very strong effect at relatively low baseline mortality (ref. 8, Fig. 1).

Our modeling approach assumes that mortality-risk variations due to location or age are interchangeable with respect to the azithromycin effect. If the assumption does not hold, then the similar effect magnitudes observed in MORDOR among 3 to 4-year-olds in Niger and infants in the other two sites are due to chance variations, or to other factors unrelated to baseline mortality.

Although using data from within MORDOR for baseline-risk estimation may seem attractive at face value, such estimates are almost inevitably subject to endogeneity bias. Our approach made sure this bias is conservative, which may explain the attenuated interaction estimate compared with the models using external data. We deem the DHS and MICS estimates at least as reliable for addressing the question of interest. These estimates were derived completely independently of MORDOR, via an established survey approach widely considered a gold standard. The IHME mortality estimates use the very same DHS and MICS surveys as their main mortality data source. Institute of Health Metrics and Evaluation 5 × 5 km grid estimates are also informed by survey household locations, cross-validated regression models using additional geographical variables, and spatial smoothing (ref. 6, Supplemental Material). Individual grid-cell estimates may be less precise in some locations, but our approach population-averages them over hundreds of grid points in each site, and is therefore considerably more robust.

MORDOR’s endpoint is all-cause mortality, but azithromycin should not have a substantial effect on noninfectious or viral infectious deaths. Therefore, the statistical tool of an interaction model is only a blunt proxy to any possible effect modification etiology. This also means that interaction models are an inherently conservative approach to detecting effect modification in this context. As to the etiology itself, the most direct explanation for a baseline-mortality effect modification is that higher child mortality rates are usually driven by a higher proportion of infectious deaths, especially after the first month of life.^10^ In the absence of evidence for association of the azithromycin effect with specific diseases, this is probably also our best explanation.

From a programmatic perspective, even with no effect modification, more lives per dose would be saved in higher mortality populations. Therefore, the moderate evidence for a disproportionately stronger effect in those populations may provide further justification for prioritizing them. In this context, our analysis views “populations” as defined by both location and age: children of different ages in the same location face very different baseline risks. Therefore, the plans already underway to corroborate the effect among infants via additional clinical trials that leverage existing delivery schemes, for example, routine immunization systems, should be accelerated. Such trials should be implemented also in some regions resembling the Malawi and Tanzania sites rather than only in the highest mortality regions. It should be noted that postneonatal infants experience a mortality risk of > 15/1,000 py across a far greater part of sub-Saharan Africa than older children.

With these suggestions in mind, the overarching goal must be for all children to enjoy a sufficiently healthy childhood in good living conditions, so that they will not need antibiotic prophylaxis to help them survive. Azithromycin can save many lives in the short and medium term, and should be viewed as a bridge for safer and faster transition to better conditions.

## Supplementary Material

Supplemental material

## Footnotes

† ClinicalTrials.gov Identifier: NCT02047981.
