# Supplementary material for: Effect Modification by Baseline Mortality in the MORDOR Azithromycin Trial
Source: Am J Trop Med Hyg. 2019 Feb 7;103(3):1295–300. doi: 10.4269/ajtmh.18-1004 (PMC7470539; doi:10.4269/ajtmh.18-1004)
Supplement: Supplementary file 1 [file tpmd181004.SD1.doc]

Supplement to Oron et al.

**Details of Individual History Reconciliation.**

Some children were coded as unknown, moved or deceased during Phase 1 or 2, but appeared again with the same individual key in the same cluster 2 or 3 visits later. In such cases, if the new reported age and gender were not discrepant with the earlier records, the child’s status was changed to having survived the intervening time. However, they were still considered missing from the intervening Phases’ census. Conversely, in case of age/gender discrepancy, the original status was retained, and a new individual key created for the newer records. Age discrepancy thresholds were based upon the distribution of discrepancies between newer ages and time-progressed ages from previous Phases, among participants with complete records. Complete records were also examined for age discrepancy in a similar manner. Reported ages discrepant with both preceding and subsequent Phases (i.e., “sandwiched” between concordant ages), were adjusted accordingly. Otherwise, cases with strong age discrepancy between consecutive visits and no clear resolution, were also split into separate individual keys. Lastly, records of children 12 months old or younger with ages identical to or younger than reported in the previous Phase, were time-progressed from the previously reported age. All in all, out of 277430 original individual keys, the records of about 16069 children (5.8%) with intervening missing Phases were reconciled, 2634 new individual keys were created (0.9%), and 23 deaths (0.5% of deaths) were re-assigned as not having occurred. Out of 730,000 individual child-Phase records, 2454 ages were time-progressed (0.3%). There were no gender discrepancies in the data.

**A few additional model details.**

We also examined incorporating location to the estimates from IHME and from within MORDOR, thus providing localized baseline-risk surfaces. However those tended to be more erratic, and the overall conclusions regarding effect modification were nearly identical. Therefore, they were not followed through to complete models, and are not presented.

For calculation of the baseline risk covariate using MORDOR data, a naïve approach would use placebo data only, as seen in Fig. 2. However, this induces a regression-to-the-mean bias in the same direction of the observed interaction effect, i.e., it is anti-conservative.[[1]](#endnote-2) We examined two ways to work around this: one still uses only the placebo arm, but as a predictor for each child’s next Phase in the trial. This means that Phase 1 (comprising 28% of mortality events) is excluded from the main model. The other approach is to incorporate both arms, which is generally conservative rather than anti-conservative, but loses less power than excluding an entire Phase. This is the approach presented in the article.

Individual entries with unknown survival status were excluded from the model. The model population was intent-to-treat, including all children enumerated during Phase-start visits, rather than only those receiving the azithromycin or placebo. Gender was not included as a covariate, since earlier separate analyses indicated it is neither a significant risk factor nor an effect modifier in this trial (A.P. Oron, report to WHO committee on azithromycin).

The uncertainty in baseline-mortality values was accounted for, as suggested by recent methodological studies, by assuming they are missing but with a known expectations and standard errors.[[2]](#endnote-3) The full model was fitted B=400 times, each time drawing baseline-mortality values out of distributions with said expectations and standard errors (which were different for different observations, depending upon child location and age). The final effect point estimate is an average of the B point estimates. The effect’s standard error (SE) *s* is calculated using the standard multiple-imputation formula


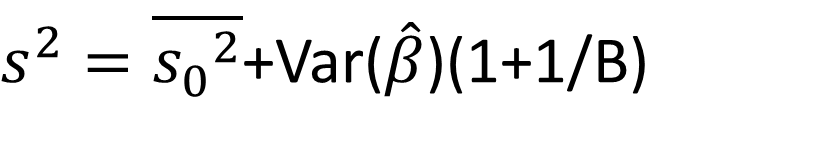
,

where the first term on the right-hand side is the average of SE estimates from the individual imputed runs, and the second term the variance of the point estimate across all imputations, multiplied by an “out-of-sample” factor.

For inference, we opted not to use permutation p-values as was done in MORDOR’s main article.[[3]](#endnote-4) The reason is that for effect modification models in a clinical trial, permuting the arm labels tests the null hypothesis *β_a_=β_mod_=0*, rather the null we aim to test which is only *β_mod_=0* without constraining *β_a_* (the main azithromycin effect). There is no standard permutation test at present for interaction terms in a survival model.[[4]](#endnote-5)

REFERENCES

1. Barnett AG, van der Pols JC, Dobson AJ. Regression to the mean: what it is and how to deal with it. *Int J Epidemiology* 2005; 34; 215–220. [↑](#endnote-ref-2)
2. Blackwell M, Honaker J, King G. A Unified Approach to Measurement Error and Missing Data: Overview and Applications. *Soc. Meth. & Res.* 2017; 46(3); 303-341. [↑](#endnote-ref-3)
3. Keenan JD, Bailey RL, West SK, et al. Mass azithromycin distribution for reducing childhood mortality in sub-Saharan Africa. *N Engl J Med* 2018; 378: 1583–92. [↑](#endnote-ref-4)
4. Foster JC, Nan B, Shen L, Kaciroti N, Taylor JM. Permutation Testing for Treatment-Covariate Interactions and Subgroup Identification. *Stat Biosci*. 2015; 8(1); 77-98. [↑](#endnote-ref-5)
